# Predicting for activity of second-line trastuzumab-based therapy in her2-positive advanced breast cancer

**DOI:** 10.1186/1471-2407-9-367

**Published:** 2009-10-17

**Authors:** Rupert Bartsch, Catharina De Vries, Ursula Pluschnig, Peter Dubsky, Zsuzsanna Bago-Horvath, Simon P Gampenrieder, Margaretha Rudas, Robert M Mader, Andrea Rottenfusser, Christoph Wiltschke, Michael Gnant, Christoph C Zielinski, Guenther G Steger

**Affiliations:** 1 Department of Medicine 1 and Cancer Centre, Clinical Division of Oncology, Medical University of Vienna, Vienna, Austria; 2 Department of Surgery, Medical University of Vienna, Vienna, Austria; 3 Department of Pathology, Medical University of Vienna, Vienna, Austria; 4 Department of Radiotherapy, Medical University of Vienna, Vienna, Austria

## Abstract

**Background:**

In Her2-positive advanced breast cancer, the upfront use of trastuzumab is well established. Upon progression on first-line therapy, patients may be switched to lapatinib. Others however remain candidates for continued antibody treatment (treatment beyond progression). Here, we aimed to identify factors predicting for activity of second-line trastuzumab-based therapy.

**Methods:**

Ninety-seven patients treated with > 1 line of trastuzumab-containing therapy were available for this analysis. Her2-status was determined by immunohistochemistry and re-analyzed by FISH if a score of 2+ was gained. Time to progression (TTP) on second-line therapy was defined as primary study endpoint. TTP and overall survival (OS) were estimated using the Kaplan-Meier product limit method. Multivariate analyses (Cox proportional hazards model, multinomial logistic regression) were applied in order to identify factors associated with TTP, response, OS, and incidence of brain metastases. *p *values < 0.05 were considered to indicate statistical significance.

**Results:**

Median TTP on second-line trastuzumab-based therapy was 7 months (95% CI 5.74-8.26), and 8 months (95% CI 6.25-9.74) on first-line, respectively (n.s.). In the multivariate models, none of the clinical or histopthological features could reliably predict for activity of second-line trastuzumab-based treatment. OS was 43 months suggesting improved survival in patients treated with trastuzumab in multiple-lines. A significant deterioration of cardiac function was observed in three patients; 40.2% developed brain metastases while on second-line trastuzumab or thereafter.

**Conclusion:**

Trastuzumab beyond progression showed considerable activity. None of the variables investigated correlated with activity of second-line therapy. In order to predict for activity of second-line trastuzumab, it appears necessary to evaluate factors known to confer trastuzumab-resistance.

## Background

Human epidermal growth factor receptor (Her) 2 (c-erb-B2) is a member of the Her-family of transmembrane receptor proteins [1]. As no ligand has been identified, Her2 is believed to act mainly via amplification of signals from other members of the Her-family (EGFR, Her3, Her4) by forming heterodimers [2]. Key proteins involved in Her2 signal-transduction include phosphatidyl-inositol (PI) 3 kinase and the ras/raf cascade. Ultimately, activation of those signalling pathways results in changes of growth, differentiation, adhesion, apoptosis and angiogenesis [3]. Her2 is overexpressed in approximately 15 - 20% of breast cancer cases, and several studies have shown that this confers a more aggressive course of disease [4,5].

Trastuzumab (rhMab4D5) is a recombinant monoclonal humanized antibody targeting the extracellular domain of Her2. Different mechanisms of action have been suggested. Trastuzumab inhibits downstream signalling pathways and blocks the shedding of Her2's extracellular domain. It causes internalization and degradation of the Her2 receptor protein, cell cycle arrest due to decreased cyclin-dependent kinase-2 (CDK2) activity via p27 induction, and inhibition of DNA repair. Antibody dependent cellular cytotoxicity (ADCC) apparently also plays a role [6,7]. In addition, trastuzumab may sensitize tumour cells to the cytotoxic effects of conventional chemotherapy [8].

Phase II clinical trials established the activity of trastuzumab as single-agent in Her2-positive metastatic breast cancer [9,10]. Randomized studies proved the combination of trastuzumab and taxanes superior in terms of response, progression-free and overall survival over chemotherapy alone [11,12]. Accordingly, trastuzumab was approved as first-line treatment of Her2-positive metastatic breast cancer in combination with taxanes. However, primary resistance may occur and many tumours who have initial response to trastuzumab will acquire secondary resistance within one year. Trastuzumab resistance is likely multi-factorial, and no clinical surrogate is yet available [7].

Upon progression on trastuzumab-based first-line treatment, two options exist. Patients may be switched to lapatinib, a tyrosine-kinase inhibitor of EGFR and Her2, alternatively they may continue on trastuzumab in combination with capecitabine. Up until recently the latter approach was supported by data from retrospective analyses, prospective observations, and a small phase II trial only [13-15], while other studies questioned the potential benefit of treatment beyond progression [16]. In the meantime, a randomized phase III study reported results, suggesting that trastuzumab in combination with capecitabine is more active than chemotherapy alone upon progression on trastuzumab-based first-line treatment in terms of response rate and progression-free survival [17]. In contrast, in a large randomized phase III study, lapatinib plus capecitabine was active upon trastuzumab failure, and patients on lapatinib had a significantly lower incidence of brain metastases [18].

As of now, there are no means to prospectively define the optimal treatment approach for the individual patient. Therefore, we aimed to identify factors predicting for efficacy of trastuzumab treatment beyond disease progression. Furthermore, we tried to identify characteristics associated with early development of brain metastases, as this population will potentially benefit most from lapatinib.

## Methods

All patient data were collected at the Department of Medicine 1 and Cancer Centre, Clinical Division of Oncology, at the Medical University of Vienna, Vienna, Austria. This retrospective analysis was performed in accordance with the ethical regulations of the Medical University of Vienna.

### Patients

Ninety-seven consecutive patients treated from 2001 until 2008 with a minimum of two lines of trastuzumab-based therapy for metastatic disease were available for this analysis. All patients were suffering from histologically confirmed Her2-positive advanced breast cancer as defined per immunohistochemistry (IHC 3+) or fluorescence in-situ hybridization (FISH +). For baseline staging evaluations all patients had CT-scan of the chest and abdomen, mammography, and gynaecologic examination. Before initiation of trastuzumab treatment, echocardiography was mandatory, and patients with left ventricular ejection fraction (LVEF) below 50% were excluded.

### Treatment plan and patient evaluation

Trastuzumab was administered at a dose of 8 mg/kg body weight loading dose on the first day of treatment, followed by 6 mg/kg body weight every three weeks thereafter [19]. Re-evaluation of patients' tumour status was performed every three cycles of treatment with CT-scan of the chest and abdomen with additional work up if indicated. Echocardiography was repeated every 6 months or immediately if symptoms of congestive heart failure occurred. If a significant LVEF drop (> 10%) was observed but LVEF remained > 50%, those intervals were shortened to every 4 weeks according to our institution standards [20].

### Statistical analysis

Time to disease progression (TTP) on the second trastuzumab-based regimen for metastatic disease was defined as primary study endpoint. Secondary endpoints consisted of response rate, time to progression on first-line trastuzumab-based therapy, cardiac toxicity, incidence of brain-metastases, and overall survival (OS). TTP was defined as the interval from the first day of treatment until documented tumour progression. If a patient died without proper restaging, TTP was measured to the first day of clinical deterioration. OS was defined as the interval from the first day of trastuzumab application until death of any cause.

According to UICC criteria, complete response (CR) was defined as disappearance of all measurable lesions for a minimum of eight weeks. Partial response (PR) was defined as 25% or more reduction in sum of products of the greatest diameters of measurable lesions, no increase of lesion size and no new lesions. Stable disease (SD) was defined as less than 25% decrease and less than 25% increase without the appearance of new lesions. Progressive disease (PD) was defined as greater than 25% increase in tumour size or the appearance of new lesions.

TTP and OS were estimated using the Kaplan-Meier product-limit method. To test for differences between TTP curves, the log-rank test was used. *p *values less than 0.05 were considered to indicate statistical significance. In the univariate analysis of TTP, the following variables were included: age > 65 years; age < 35 years; tumour stage at primary diagnosis (localized versus metastatic); grading (1,2 versus 3); histological subtype (ductal versus lobular carcinoma); hormone receptor status (oestrogen and/or progesterone receptor positive versus negative); metastatic sites (non-visceral only versus visceral involvement); number of metastatic sites (1 versus ≥ 2; ≤ 2 versus > 2); time to disease recurrence < 12 months following primary treatment; trastuzumab from diagnosis of metastatic disease; response (CR + PR) to first-line trastuzumab treatment; and appearance of new metastatic sites upon progression on first-line trastuzumab. In the analysis of factors predicting for OS, development of brain metastases as well as early development of brain metastases (< 12 months after initiation of trastuzumab) were also included.

A Cox proportional hazard model was applied as multivariate analysis to evaluate factors associated with OS and TTP on second-line trastuzumab-based therapy. Variables exhibiting significance (p < 0.05) or near significance (p < 0.08) at univariate analysis were included into the Cox regression models. To evaluate variables associated with treatment response to second-line trastuzumab-based therapy as well as development of brain metastases, a multinomial logistic regression model was used.

All statistics were calculated using statistical package for the social sciences (SPSS^®^) 16.0 software (SPSS Inc., Chicago, IL, USA). Data were analyzed as of February 2009.

## Results

### Patient characteristics

Ninety-seven consecutive patients, all female, suffering from Her2-positive metastatic breast cancer were identified from a breast cancer database. Median age was 51 years, range 25-78 years. Table 1 lists the characteristics of all patients included. 83.5% had invasive ductal carcinoma; 42.3% had positive hormone receptor status, and 66% grade 3 tumours. Sixty-seven patients (69.1%) were treated with adjuvant chemotherapy. Adjuvant endocrine therapy was administered in 26 patients (26.8%), and a further 9.3% received adjuvant trastuzumab for a median duration of 10 months (range 4-24 months). Twenty-four patients (24.7%) had metastatic disease at the time of primary diagnosis. Median time to disease recurrence in the reminders was 24.5 months (range 4-210 months). In 75.3%, visceral metastases were present upon initiation of palliative trastuzumab.

**Table 1:** Patient characteristics

Characteristics	Patients
Entered	n = 97

Median age (years) (range)	46 (25-73)

Age > 65 years	12 (12.4%)

Age < 35 years	8 (8.2%)

Stage 4 at primary diagnosis	24 (24.7%)

Grade 3 tumour	64 (66%)

Invasive ductal carcinoma	81 (83.5%)

Positive hormone receptor status	41 (42.3%)

Visceral metastases	73 (75.3%)

Median metastatic sites (sites) (range)	2 (1-6)

Lung	36

Liver	49

Bones	51

Lymph nodes	37

Soft tissue	48

Skin	16

Brain (before trastuzumab)	1

Others	3

More than one metastatic site	78 (80.4%)

Adjuvant chemotherapy	67 (69.1%)

Anthracyycline based	37

Taxane based	2

Combination anthracyclines/taxanes	22

Adjuvant endocrine therapy	26 (26.8%)

Adjuvant trastuzumab	9 (9.3%)

Duration adjuvant trastuzumab (median) (range)	10 (4-24)

Palliative treatment other than trastuzumab-based	35 (36.1%)

Second-line combination	

Taxanes	21

Vinorelbine	30

Capecitabine	23

Gemcitabine	12

Anthracyclines	5

Others	6

Trastuzumab beyond second-line	55 (56.7%)

Lapatinib beyond second-line trastuzumab	14 (14.4%)

36.8% had received prior non-trastuzumab-containing therapy for metastatic disease, and 14.4% were switched to lapatinib after a median of four (range 2-9 lines) trastuzumab-based treatment lines.

### Response and survival data

Follow-up data is available on all but one patient. Median time of observation was 24 months (range 8-84 months). Median TTP (second-line trastuzumab-based therapy) was 7 months (95% CI 5.74-8.26), and 8 months (95% CI 6.25-9.74) on first-line trastuzumab-based therapy respectively (n.s.). Clinical benefit rate (CBR) in the second-line setting was 66% (CR 7.2%, PR 23.7%, SD > 6 months 35.1%), as compared to CBR 83.5% (CR 9.3%, PR 35.1%, SD > 6 months 39.2%) on first-line. Efficacy endpoints are summarized in Table 2.

**Table 2:** Efficacy endpoints

Endpoint	Results
Median time to progression second-line trastuzumab-based therapy (months)	7 (95% CI 5.74-8.26)

Median time to progression first-line trastuzumab-based therapy (months)	8 (95% CI 6.26-9.74)

Overall survival (months)	43 (95% CI 37,92-48.09)

Response rate second-line trastuzumab-based therapy (%)	30.9%

Complete response (%)	7.2%

Partial response (%)	23.7%

Response rate first-line trastuzumab-based therapy (%)	44.3%

Complete response (%)	9.3%

Partial response (%)	35.1%

Clinical Benefit second-line trastuzumab-based therapy (%)	66%

Clinical Benefit first-line trastuzumab-based therapy (%)	83.5%

Time to development of brain metastases (months)	21 (95% CI 13.86-28.14)

Overall survival after treatment for brain metastases (months)	10 (95% CI 4.53-15.47)

Drop of left ventricular ejection fraction (LVEF) > 10% (n=)	3

Symptomatic congestive heart failure (n=)	2

Patients with more than one metastatic site (TTP median 6 months [95% CI 5.06-6.98] versus 14 months [95% CI 9.56-18.44]; p = 0.013) as well as those with more than two metastatic sites (TTP median 6 months [95% CI 5.09-6.91] versus 8 months [95% CI 5.58-10.42]; p = 0.003) exhibited a significantly shorter time to disease progression (Table 3). Therefore, one versus more than one metastatic site appeared superior in prediction for TTP on second-line trastuzumab-based therapy and was included into the multivariate model. Furthermore, patients younger than 35 years derived less benefit from a second trastuzumab-based treatment line (TTP median 3 months [95% CI 0.23-5.77] versus 7 [95% CI 5.67-8.33]; 0.026) (Table 3). In the multivariate model however, only one versus more than one metastatic site was significantly associated with TTP (p = 0.037, OR 1.91, 95% CI 0.25-1.12) (Table 4).

**Table 3:** Univariate analysis: Influence on TTP (second-line)

Factor	Median TTP (months)	95% CI	SE*	p =
Age > 65 years	6 vs. 7	0.00-12.79	3.36	n.s.
		5.64-8.36	0.7	

Age < 35 years	3 vs. 7	0.23-5.77	1.41	0.026
		5.67-8.33	0.68	

Stage 4 at diagnosis	6 vs. 7	5.52-8.48	0.76	n.s.
		0.39-11.61	2.86	

Grading (1,2 versus 3)	6 vs. 7	2.98-9.03	1.54	n.s.
		5.6-8.41	0.72	

Histologic type (ductal versus lobular)	6 vs. 9	4.86-7.14	0.58	n.s.
		0.71-17.29	4.23	

Positive hormone receptor status	6 vs. 7	4.64-7.36	0.7	n.s.
		5.14-8.86	0.95	

Time to recurrence < 12 months	6 vs. 7	3.41-8.59	1.32	n.s.
		5.61-8.39	0.71	

Visceral metastases	7 vs. 6	5.76-8.25	0.64	n.s.
		0.00-12.53	3.33	

Number of metastatic sites (1 versus 2)	14 vs. 6	9.56-18.44	2.26	0.013
		5.02-6.98	0.5	

Number of metastatic sites (2 versus > 2)	8 vs. 6	5.58-10.42	1.23	0.003
		5.09-6.91	0.47	

Trastuzumab from first-line palliative treatment	7 vs. 6	5.62-8.38	0.71	n.s.
		4.65-7.35	0.69	

Response to first-line trastuzumab-based therapy	7 vs. 6	5.25-8.75	0.89	n.s.
		3.77-8.23	1.14	

New metastatic sites at progression upon first-line trastuzumab-based therapy	7 vs. 6	4.81-9.19	1.12	n.s.
		5.12-6.88	0.45	

* SE: Standard error

**Table 4:** Cox proportional hazard model: Prediction of TTP (second-line)

Factor	p =	OR*	95% CI
Age < 35 years	0.097	0.53	1.04-3.5

Number of metastatic sites (1 versus 2)	0.037	1.91	0.25-1.12

* OR: Odds ratio

None of the variables could independently predict for response to second-line trastuzumab-based therapy in the multinomial logistic regression model (Table 5).

**Table 5:** Multinomial logistic regression model: Prediction of Response (second-line)

Factor	p =	OR	95% CI
Age > 65 years	0.392	0.35	0.03-3.9

Age < 35 years	0.363	0.3	0.02-4

Stage 4 at diagnosis	0.839	1.11	0.4-3.14

Grading (1,2 versus 3)	0.268	0.37	0.06-2.15

Histologic type (ductal versus lobular)	0.19	0.17	0.01-2.41

Positive hormone receptor status	0.534	0.63	0.15-2.68

Time to recurrence < 12 months	0.332	0.43	0.08-2.36

Visceral metastases	0.147	3.96	0.62-25-35

Number of metastatic sites (1 versus 2)	0.318	0.36	0.05-2.64

Number of metastatic sites (2 versus > 2)	0.792	0.82	0.18-3.63

Trastuzumab from first-line palliative treatment	0.886	1.15	0.18-7.53

Response to fist-line trastuzumab-based therapy	0.201	2.58	0.6-11-08

New metastatic sites at progression upon first-line trastuzumab-based therapy	0.953	1.04	0.28-3.84

One patient had brain metastases before initiation of antibody therapy, and thirty-nine (40.2%) developed brain metastases either on second-line treatment or thereafter. Median time to development of brain metastases was 21 months (95% CI 13.86-28.14), and overall survival following local treatment for brain metastases was 10 months (95% CI 4.53-15.47).

Twelve patients (30.8%) developed brain metastases in less than one year. In this subgroup, early development of brain metastases was significantly associated with the presence of visceral metastases in a multinomial logistic regression model (p = 0.012).

**Figure 1:**
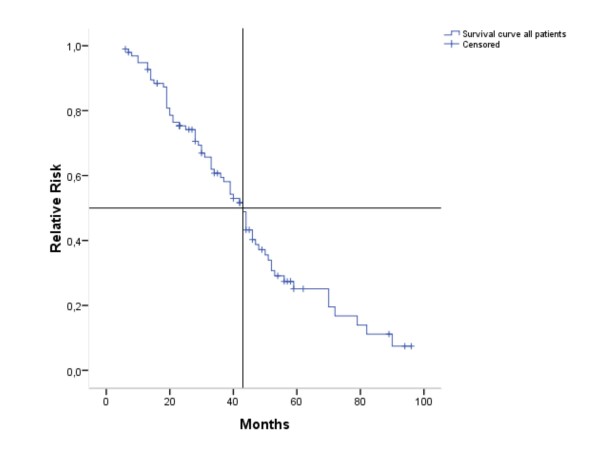
**Kaplan Meier estimation of overall survival (months)**. n = 97

OS in the entire population was 43 months (95% CI 37.92-48.09) (Figure 1). In the univariate analyses (log-rank test), the following variables were significantly or near significantly associated with shorter OS: Age younger than 35 years (p = 0.023), hormone receptor negative disease (p = 0.075), more than one metastatic site (p = 0.003), more than two metastatic sites (p = 0.010), development of brain metastases (p = 0.010) as well as early development of brain metastases (p < 0.001) (Table 6). In the Cox regression model, only early development of brain metastases (p = 0.001) retained significance (Table 7).

**Table 6:** Univariate analysis: Influence on OS

Factor	Median TTP (months)	95% CI	SE	p =
Age > 65 years	44 vs. 43	29.77-58.23	7.26	n.s.
		37.75-48.25	2.68	

Age < 35 years	25 vs. 44	7.32-42.68	9.02	0.023
		38.5-49.5	2.81	

Stage 4 at diagnosis	43 vs. 39	37.74-48.26	2.69	n.s.
		25.96-52.04	6.66	

Grading (1,2 versus 3)	43 vs. 43	31.94-54.06	5.64	n.s.
		37.38-48.72	2.92	

Histologic type (ductal versus lobular)	43 vs. 52	38.35-47.65	2.37	n.s.
		8.41-95.6	22.24	

Positive hormone receptor status	47 vs. 39	38.4-55.61	4.39	0.075
		31.95-46.05	5.6	

Time to recurrence < 12 months	46 vs. 42	41.52-50.48	2.29	n.s.
		35.7-48.3	3.21	

Visceral metastases	40 vs. 53	33.82-46.18	3.16	n.s.
		42-62	5.1	

Number of metastatic sites (1 versus 2)	NR* vs. 37	na^§^	na^§^	0.003
		27.35-46.65	4.92	

Number of metastatic sites (2 versus > 2)	46 vs. 33	35.24-56.73	5.49	0.010
		19.56-46.44	6.86	

Trastuzumab from first-line palliative treatment	43 vs. 33	38.65-47.35	2.2	n.s.
		9.02-57.98	12.75	

Response to first-line trastuzumab-based therapy	46 vs. 42	36.33-55.67	4.94	n.s.
		34.5-49.5	3.82	

New metastatic sites at progression upon first-line trastuzumab-based therapy	44 vs. 42	26.05-61.96	9.16	n.s.
		29.69-54.31	6.38	

Development of brain metastases	33 vs. 46	32.81-43.19	2.9	0.010
		40.32-51.69	5.2	

Early development of brain metastases	19 vs. 44	9.11-28.89	2	< 0.001
		40.07-47.93	5.05	

* NR: Median OS not reached

^§ ^na: Not available

**Table 7:** Cox proportional hazard model: Prediction of OS

Factor	p =	OR	95% CI
Age < 35 years	0.083	2.1	0.91-4.86

Positive hormone receptor status	0.075	0.61	0.36-1.05

Number of metastatic sites (1 versus 2)	0.053	4.2	1.74-1.01

Development of brain metastases	0.292	1.35	0.77-2.37

Early development of brain metastases	0.001	3.83	1.68-8.71

### Cardiac Toxicity

As outlined above, echocardiography was performed every six months longest, with shorter intervals if indicated. A significant drop in LVEF was observed in three patients (3.1%); in one patient, trastuzumab had to be stopped temporarily and in another discontinued permanently due to symptomatic congestive heart failure (Table 2).

In none of the patients switched to lapatinib, a significant drop in LVEF or congestive heart failure was observed.

## Discussion

Concerning further therapy upon progression on trastuzumab-based first-line treatment, two options are available: Continuation of trastuzumab plus capecitabine, or a switch to the tyrosine kinase inhibitor lapatinib, again in combination with capecitabine. Both strategies are supported by data from prospective randomized phase III trials [17,18], although a continuation of trastuzumab is potentially less well established, as the trial conducted by the German Breast Group (GBG-26) had to be closed early due to poor accrual [17]. Still, given those results as well as data from a number of smaller studies, it is safe to assume that there is a benefit from trastuzumab beyond progression in a subset of patients. Therefore, identifying this group in order to optimize palliative treatment for the individual patient is important.

Our retrospective data again suggest that continuation of trastuzumab is a valuable salvage option for patients with advanced Her2-positive breast cancer, whose disease has progressed on prior trastuzumab-based regimens. TTP on trastuzumab-based second-line treatment was 7 months median, and therefore not significantly different from the first-line setting. Those data are well in line with results from different other studies as well as the single phase III trial. In those studies, a TTP of 6 - 8 months on second-line trastuzumab-based therapy was reported [13-17].

OS in our population was 43 months. This is considerably longer than survival reported in the pivotal trastuzumab plus taxane trials [11,12], and might even hint at a survival-benefit associated with the use of trastuzumab in multiple lines. Still, as this is a retrospective analysis, data need to be interpreted with caution: Only patients who had at least two lines of trastuzumab-based therapy were included. Outcome for patients with rapid progression, not eligible for second-line trastuzumab-based treatment, therefore is not reported. This potential bias might well add to the magnitude of the observed survival advantage.

Our study was initiated in order to identify readily available clinical or histopathological factors potentially predicting for activity of trastuzumab treatment beyond progression. Furthermore, we tried to establish risk factors for early development of brain metastasis, as this subgroup might derive the largest benefit from lapatinib due to the fact that tyrosine-kinase inhibitors may pass an intact blood-brain-barrier. In the trial conducted by Geyer et al, this assumption was proven correct by the significantly lower number of brain metastases observed in the lapatinib group [18].

In the Cox regression model, only the number of metastatic sites (one versus more than one) was significantly associated with time to disease progression on second-line trastuzumab-based therapy. Trastuzumab therefore seemed to have higher activity in patients with a low number of metastases. While this could indicate a higher responsiveness of certain tumours to trastuzumab, it is rather possible that this result would have been observed with lapatinib or chemotherapy alone as well. In the multinomial logistic regression model none of the factors analysed predicted for response to second-line trastuzumab-based therapy.

Therefore, readily available clinical and histopathological features are not sufficient as decision-making tool, and further exploration of mechanisms of resistance against trastuzumab is warranted. Patients should be tested for truncated Her2, as trastuzumab cannot bind to Her2-molecules lacking the extracellular domain [7]. Furthermore, two recently presented studies suggested that tumours with PTEN-loss and activated PI3-Kinase pathways may be less responsive to trastuzumab [21,22].

As for overall survival, early development of brain metastases predicted for inferior outcome. In a logistic regression model, development of brain metastases in less than 12 months was significantly associated with presence of visceral metastases. This however is a feature of most Her2-positive tumours, therefore, this subgroup awaits further specification.

## Conclusion

In conclusion, this retrospective analysis indicates that trastuzumab has increased overall survival in Her2-positive patients. Furthermore, those results again suggest that a subgroup of patient may benefit from trastuzumab-treatment in multiple lines. On the other hand, we were not able to identify clinical or histopathological markers predicting for activity of this approach. Therefore, identification of other biomarkers of trastuzumab resistance is urgently warranted.

## Competing interests

Rupert Bartsch has received lecture honoraria from Glaxo Smith Kline and Hoffmann-La Roche. Michael Gnant has received lecture honoraria from Hoffmann-La Roche. Christoph C. Zielinski has received lecture honoraria from Hoffmann-La Roche. Guenther G. Steger has received lecture honoraria from Glaxo Smith Kline and Hoffmann-La Roche. No further potential conflicts of interest are to be disclosed.

## Authors' contributions

RB contributed the idea for this trial, participated in the design and drafted the manuscript, CD participated in the statistical analysis and helped drafting the manuscript, UP and CW both participated in the collection of patient data, PD and AR helped in the design and coordination of the study, ZB and MR did the immunohistochemical staining and FISH analyses, SG recorded patient data, RM assisted in the statistical analysis, MG revised the manuscript critically, CZ and GS participated in the design of the study, the coordination and assisted with drafting the manuscript.

All authors have read and approved the final manuscript.

## Pre-publication history

The pre-publication history for this paper can be accessed here:

http://www.biomedcentral.com/1471-2407/9/367/prepub
